# Functional characterization of two defensins, HlDFS1 and HlDFS2, from the hard tick *Haemaphysalis longicornis*

**DOI:** 10.1186/s13071-017-2397-9

**Published:** 2017-10-02

**Authors:** Ta Sun, Wen Pan, Yanhui Song, Jingpin Zhang, Jingwen Wang, Jianfeng Dai

**Affiliations:** 1 Institutes of Biology and Medical Sciences, Jiangsu Key Laboratory of Infection and Immunity, Soochow University, Suzhou, People’s Republic of China; 2 Department of Clinical Laboratory, The First Affiliated Hospital of Soochow University, Suzhou, People’s Republic of China; 3 School of Life Science, Fudan University, Shanghai, People’s Republic of China

**Keywords:** Defensin, Antimicrobial peptide (AMP), Antibiotic resistant bacteria, Tick, *Haemaphysalis longicornis*

## Abstract

**Background:**

Ticks are second to mosquitoes as vectors of human arthropod-borne diseases. Ticks rely heavily on antimicrobial peptides (AMPs) to defend against microbes and defensins are major components of innate immunity in ticks.

**Results:**

Two novel defensin genes, named HlDFS1 and HlDFS2, were identified from a cDNA library of the hard tick *Haemaphysalis longicornis* collected in southeast China. The peptides encoded by both genes shares typical features of type-2 arthropod defensin superfamily. The expressions of both genes increased in ticks during blood-feeding. The synthetic minimum functional peptides HlDFS1 and HlDFS2 showed broad spectrum antimicrobial activity against various Gram-positive and Gram-negative bacteria. Moreover, HlDFS1 and HlDFS2 exhibit bactericidal activity to some drug resistant bacteria. HlDFS1, but not HlDFS2, showed inhibitory activity against fungus *Candida albicans*. HlDFS1 and HlDFS2 had no significant hemolysis effect on human erythrocytes at low concentrations and did not impair mammalian cell survival. Finally, HlDFS1 and HlDFS2 significantly protected mice against lethal infection by *Staphylococcus aureus* and *Micrococcus luteus*.

**Conclusions:**

HlDFS1 and HlDFS2 are two novel functional defensins from the hard tick *Haemaphysalis longicornis*. They showed bactericidal activity against various Gram-positive and Gram-negative bacteria and significantly protect mice against lethal bacterial infection. Thus, HlDFS1 and HlDFS2 can be introduced to the medical field as new drug candidates with antibacterial activity.

**Electronic supplementary material:**

The online version of this article (10.1186/s13071-017-2397-9) contains supplementary material, which is available to authorized users.

## Introduction

Ticks are obligate hematophagous ectoparasites, living by feeding on the blood of mammals, birds, and sometimes reptiles and amphibians [1]. Ticks harm people indirectly by transmitting pathogenic organisms, such as protozoa, viruses and bacteria. As they are widely distributed around the world, especially in warm, humid climates, ticks occupy the second place, after mosquitoes, in the abundance of transmission of arthropod-borne diseases. The typical tick-borne diseases include tick-borne encephalitis, Crimean-Congo hemorrhagic fever, Lyme disease, Q fever and Rocky Mountain spotted fever, among others [1, 2].

Lacking lymphocytes, thymuses, or antibodies, ticks rely heavily on antimicrobial peptides (AMPs) to defend against microbes, so that they can live harmoniously with microbes [3–5]. Among the identified and widely characterized tick AMPs, defensins and their isoforms have been identified in many tick species including *Amblyomma americanum*, *Dermacentor variabilis*, *Haemaphysalis longicornis*, *Ixodes scapularis*, *Ixodes ricinus*, *Ornithodoros moubata* and *Rhipicephalus microplus* [6–9]. Defensins are small cysteine-rich cationic proteins (usually contain six cysteine residues) forming disulfide bridges with the conserved pairing Cys1-Cys4, Cys2-Cys5, and Cys3-Cys6 [3, 6, 10]. They are usually expressed in the midgut (MG) after blood-feeding and pathogen invasion [11]. For examples, hemocoelic inoculations with *Borrelia burgdorferi* of *D. variabilis* induced upregulation of a lysozyme-like peptide and the secretion of defensin [12, 13]. The expressions of a 5.3-kd defensin family were upregulated upon tick acquisition of *Anaplasma phagocytophilum* [14]. Tick defensins have a broad spectrum of antimicrobial profiles, which are primarily directed against Gram-positive bacteria, but some isoforms are also effective against Gram-negative bacteria, viruses, fungi and protozoa [6, 15, 16].

In this study, we characterized two defensin genes from a cDNA library of *H. longicornis*. This hard tick species was collected in the field of Zhejiang Province, located in southeast China. Lyme spirochetes, spotted fever group rickettsiae, as well as *Ehrlichia chaffeensis* and *Anaplasma bovis*, have been detected in *H. longicornis* [17, 18]. A newly identified tick-borne virus, SFTS (Severe fever with thrombocytopenia syndrome virus, fatality rates ranging between 12 and 30% in some areas), has also been shown to be transmitted via *H. longicornis* [19, 20].

## Methods

### RNA sequencing and gene identification

Total RNA from whole adult ticks *H. longicornis* were prepared and subjected to high throughput RNA sequencing. A total of 43,419 pieces of Unigene sequences were obtained and screened for novel defensing-like genes by BLAST analyses against the NCBI NR database (https://blast.ncbi.nlm.nih.gov/Blast.cgi).

### Gene expression

RNA was extracted from whole adult ticks before and after tick feeding for 72 h on a Specific Pathogen Free (SPF) mouse. Transcript expression analysis of HlDFS1 and HlDFS2 genes was conducted by reverse transcription polymerase chain reaction (RT-PCR) using HlDFS1 and HlDFS2 gene specific primers (see primer sequences in Additional file 1: Table S1).

### Antimicrobial assay

To test the antimicrobial activity of HlDFS1 and HlDFS2, the minimum functional segments of HlDFS1 (a 37 amino acid peptide) and HlDFS2 (a 36 amino acid peptide) were synthesized and purified by high-performance liquid chromatography (the final purity of peptides are > 90%) (GL Biochem, Shanghai, China). The peptides were dissolved in PBS buffer containing 0.05% Tween 20 and 1 μM β-merkaptoethanol (at a stock concentration of 1000 μM), and diluted properly in PBS when used in the antimicrobial assay. The target bacteria used in the bactericidal assay were obtained from China Veterinary Culture Collection Center (CVCC) and operated with standard protocols. The antimicrobial assays were performed as described previously [7]. Briefly, microbial strains were grown to an OD600 nm of 0.4–0.6 at 37 °C in Poor Broth media (1% *w*/*v* tryptone and 0.5% *w*/*v* NaCl) (except for *Mycobacterium bovis*, which was grown in Middlebrook 7H9 Broth (BD-Difco, Franklin Lakes, USA) with carbenicillin, *Candida albicans* which was grown in Sabouraud’s Dextrose Broth (1% *w*/*v* tryptone, 4% w/v glucose) and *B. burgdorferi* which was grown in complete BSK media (Sigma, St. Louis, USA) [21]. Approximately 90 μl of inocula of microbial strains (diluted with PB media to an OD600 nm of 0.001) were incubated with 10 μl of various concentrations of HlDFS1 and HlDFS2 (0.1–50 μM) in wells of a 96-well plate. The mixture was grown for 20 h (for *M. bovis*, incubated for 48 h) at 37 °C and 250 rpm. Antimicrobial activity was evaluated by measuring the absorbance of the bacterial culture at 595 nm. *Borrelia burgdorferi* 297-GFP strain is an engineered spirochetes that steadily expressing GFP [22]. The anti-*borrelia* activities of HlDFS1 and HlDFS2 were analyzed by amplifying the *flaB* DNA copies in the culture media and visualizing GFP signals under a fluorescence microscope.

Eleven antibiotic resistant bacteria strains were obtained from the First Affiliated Hospital of Soochow University (Suzhou, China) under institutional guidelines. The antibiotic resistant information of some of the strains is listed in Additional file 2: Table S2. The antimicrobial activity of HlDFS1 and HlDFS2 on these antibiotic resistant bacteria were performed according to the protocols above.

### Hemolysis and cytotoxic assay

Human erythrocytes hemolysis assay was performed as previously described. 0–50 μM of HlDFS1 and HlDFS2 were incubated with human erythrocytes for 30 min at 37 °C. Red blood cells were lysised by 0.4% TritonX 100 (Sigma, St. Louis, USA) and loaded as positive controls. To test whether HlDFS1 and HlDFS2 inhibit mammalian cell proliferation, 20 μM of HlDFS1 and HlDFS2 were added into the cell media of mammalian cell line A549, 293 T, K562 and THP1 and incubated for 24 h. The cell viability assay was determined by Cell Viability Assay (Promega, Fitchburg, USA).

### Mice infection and protection assay

Mice were infected with lethal doses of *Micrococcus luteus* (1 × 10^6^ *cfu* per C3H/HeJ mouse) and *Staphylococcus aureus* (1 × 10^8^ *cfu* per C57/BL6 mouse) by intraperitoneal (i.p.) injection according to protocols described previously [23, 24]. Six h after infection, mice were treated by i.p. injection of HlDFS1 (100 μg/mouse), HlDFS2 (100 μg/mouse) or saline as controls. Mice were monitored daily for survival and symptoms of disease.

## Results and discussion

### Isolation of defensin genes HlDFS1 and HlDFS2 from *H. longicornis*

**Fig. 1:**
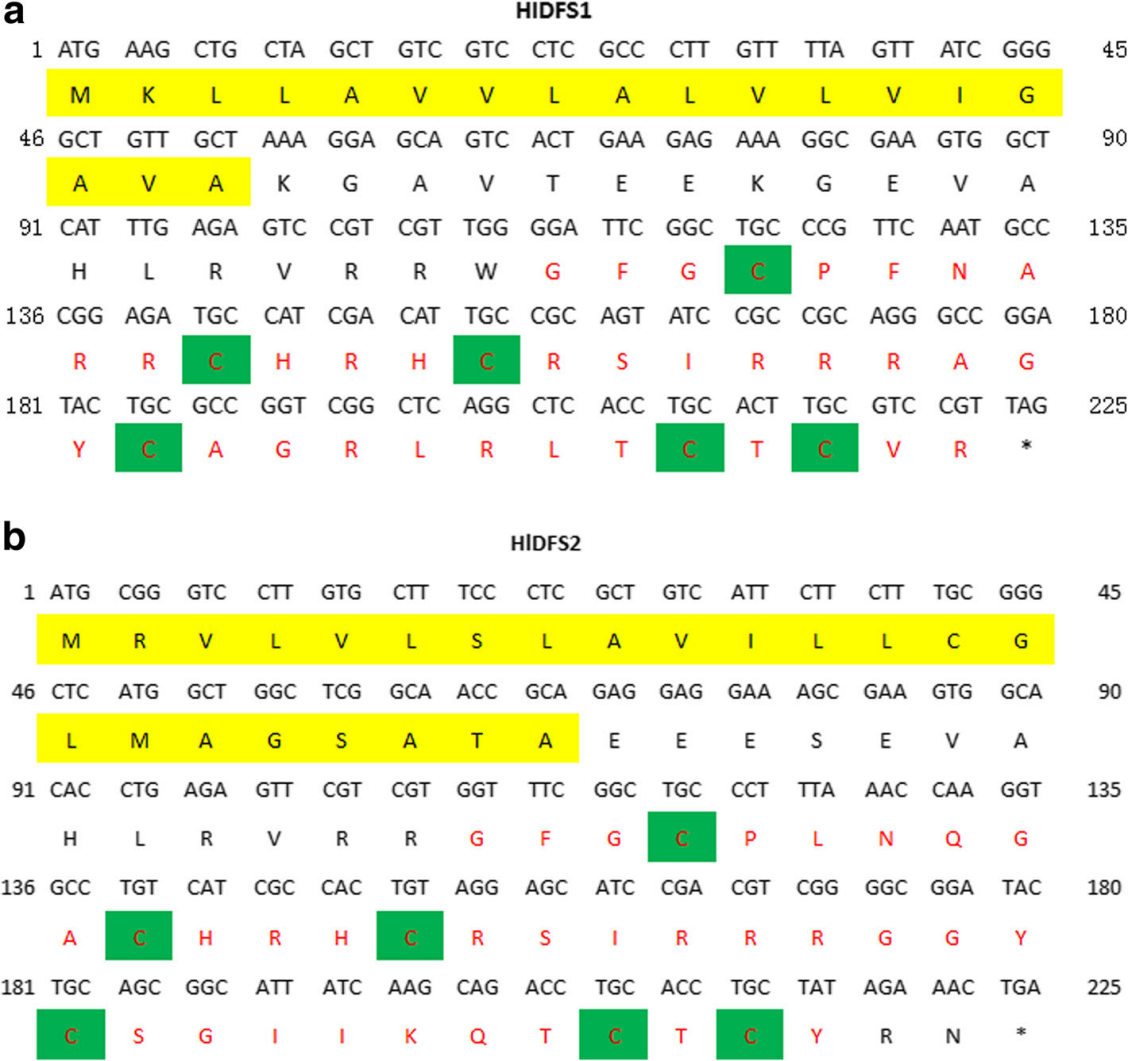
Nucleotide and deduced amino acid sequences of HlDFS1 (**a**) and HlDFS2 (**b**). The signal peptide sequence is shaded in yellow. The red lettering indicates the minimum functional segment, and the green shadow indicates the location of the conserved cysteine residues found in tick defensins. The stop codons were indicated by an asterisk

By sequencing a cDNA library of *H. longicornis*, two cDNA clones encoding the precursor of putative defensins were obtained and named as HlDFS1 and HlDFS2, respectively. The cDNA and deduced amino acid sequences of HlDFS1 and HlDFS2 are shown in Fig. 1. Sequence analysis indicated that both defensin ORFs were 225 bp long, encoding 74 amino acid peptides. The predicted HlDFS1 and HlDFS2 proteins contained a putative signal peptide cleavage site at amino acid positions 18 and 23, respectively, as analyzed by SignalP4.1 software (http://www.cbs.dtu.dk/services/SignalP/). By BlastP analysis against the protein database of NCBI (https://blast.ncbi.nlm.nih.gov/Blast.cgi), the minimal critical domains of type-2 arthropod defensins, lack of the signal peptide and leading peptides, were also predicted in both HlDFSs (Fig. 1). The sequences of HlDFS1 and HlDFS2 cDNA have been deposited in the GenBank database under the accession numbers KY113087 and KY113088.

### Homology analysis and mRNA expression of HlDFS1 and HlDFS2

**Fig. 2:**
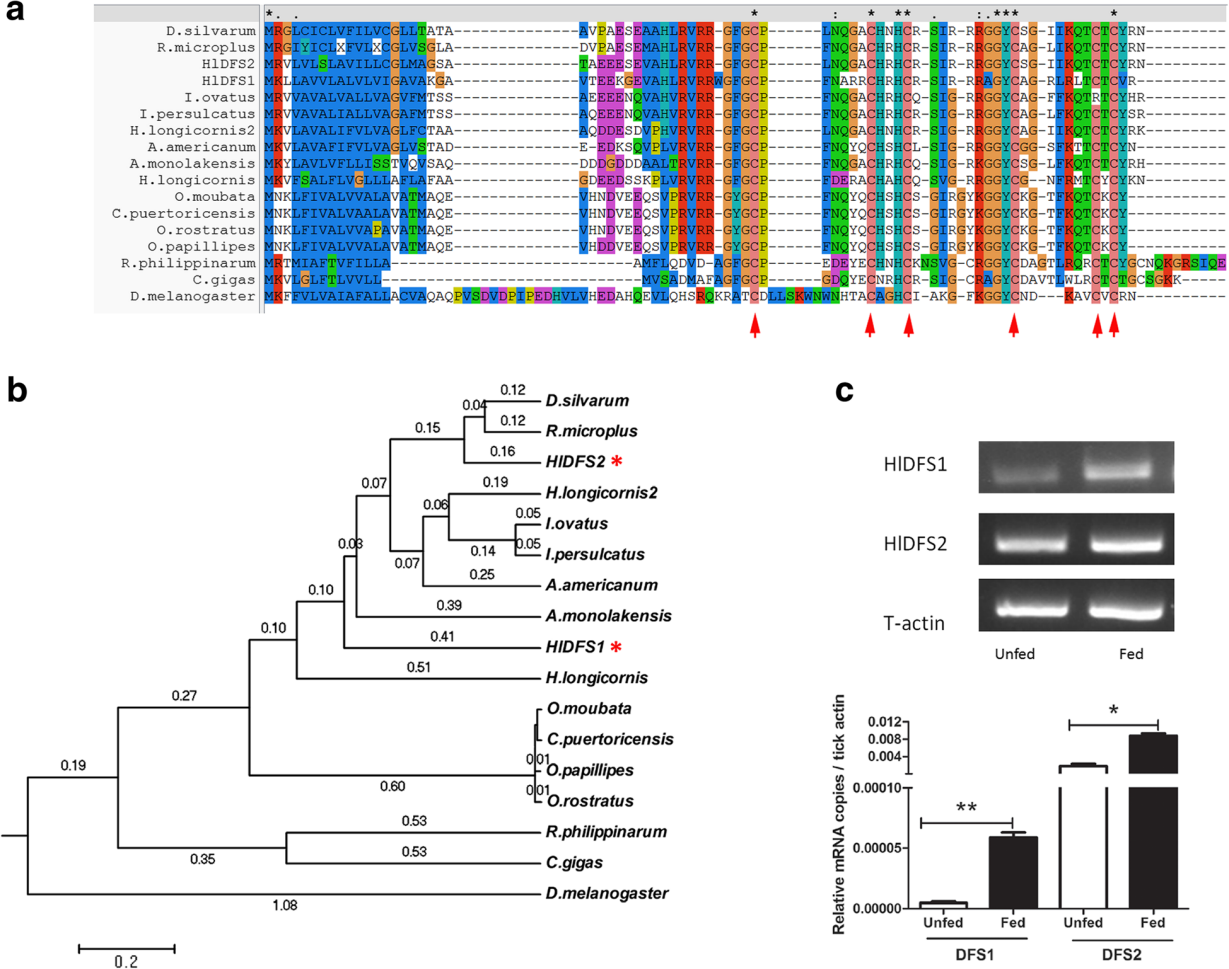
Homology analysis and mRNA expression of HlDFS1 and HlDFS2. Sequence alignment (**a**) and phylogenetic tree (**b**) constructed with sequences of defensins from *H. longicornis* and other species. HlDFS1 and HlDFS2 are indicated with red asterisks. Sequence GenBank accession numbers: *H. longicornis* 1 (ABO28925.1); *H. longicornis* 2 (BAD93183.1); *R. microplus* (AAO48943); *O. moubata* (BAB41028); *O. papillipes* (ACJ04425.1); *O. rostratus* (ACJ04428.1); *C. puertoricensis* (ACJ04429.1); *A. monolakensis* (ABI52766.1); *D. silvarum* (AJG42673.1); *I. ovatus* (BAH09305.1); *I. persulcatus* (BAH09304.1); *A. americanum* (ABI74752.1); *R. philippinarum* (ADO32580.1); *C. gigas* (ACQ76287.1): *D. melanogaster* (AAF58855.1). **c** RT-PCR (upper panel) and quantitative RT-PCR (lower panel) analysis of HlDFS1 and HlDFS2 mRNA expression in unfed and fed adult *H. longicornis*. RNA were isolated from unfed and fed (for 72 h) whole ticks, and expression level of HlDFS1 and HlDFS2 mRNA were amplified by semi-quantitative and SYBR Green quantitative PCR using gene specific PCR (Additional file 1: Table S1). Tick actin gene was amplified as the loading control. Results are expressed as the mean ± SEM. *P < 0.05 and **P < 0.01 (*t*-test). The representative results from at least three independent experiments are shown

Sequence alignment and phylogenetic analysis were conducted as described previously [7] to explore the evolutionary relationships between HlDFS1, HlDFS2 and other defensin genes in invertebrate species (Fig. 2a, b). The analysis was performed using multiple sequence alignment software ClustalW (http://www.genome.jp/tools-bin/clustalw). The results suggest that HlDFS1 and HlDFS2 shared high similarity to defensins from hard tick species, such as *H. longicornis* (*vs* BAD93183.1, 52.7% and 67.6%, respectively) *R. microplus* (65% and 73%, respectively), *D. silvarum* (54% and 77%, respectively), *I. persulcatus* (64% and 67%, respectively), *I. ovatus* (59% and 64%, respectively) and *A. americanum* (53% and 55%, respectively), and lower similarity to defensins from soft ticks, such as *O. papillipes* (45% and 47%, respectively), *O. rostratus* (45% and 46%, respectively) and *C. puertoricensis* (48% and 46%, respectively). All these defensins contained six conserved cysteine residues, including defensins from fruit fly (*Drosophila melanogaster*) or marine mollusk species (*Crassostrea gigas* and *Ruditapes philippinarum*) (Fig. 2a), suggesting these amino acids were critical for their function [9].

To investigate the mRNA expression of HlDFS1 and HlDFS2 during tick feeding, RNA was extracted from whole adult ticks before and after tick feeding on a clean mouse. Transcript expression analysis of HlDFS1 and HlDFS2 genes was conducted by quantitative RT-PCR using HlDFS1 and HlDFS2 gene specific primers (see Additional file 1: Table S1). As shown in Fig. 2c, the expression levels of HlDFS1 and HlDFS2 were significantly higher in fed ticks than in unfed ticks, suggesting they are induced during tick feeding.

### Antimicrobial profiles of HlDFS1 and HlDFS2

The target bacteria used in the bactericidal assay were obtained from China Veterinary Culture Collection Center (CVCC) and operated with standard protocols. Gram-positive bacteria, *Bacillus pumilus* (CMCC63202), *S. aureus* (CMCC26003), *M. luteus* (CMCC28001), and *M. bovis*; Gram-negative bacteria, *Salmonella typhimurium* (CVCC542), *Pseudomonas aeruginosa* (CVCC2000), *Escherichia coli* (CMCC44103), and *B. burgdorferi* (297-GFP strain); and fungus *C. albicans* (CAU0037), were used in this study. The antimicrobial assays were performed as described previously [7]. As shown in Table 1, synthetic HlDFS1 and HlDFS2 showed bactericidal activities against three Gram-positive bacteria (*M. bovis, M. luteus* and *S. aureus*). The minimal inhibitory concertation of HlDFS1 and HlDFS2 against *M. bovis* (MIC_90_ 0.5 μM and 0.5 μM, respectively) and *M. luteus* (MIC_90_ 10 μM and 1 μM, respectively) were relatively low. HlDFS1 and HlDFS2 did not affect the growth of *B. pumilus*. Unlike other defensins reported less effective to Gram-negative bacteria, HlDFS1 and HlDFS2 also had an ability to inhibit some Gram-negative bacteria, for example *E. coli* (MIC_90_ 5 μM and 1 μM, respectively) and *B. burgdorferi* (MIC_90_ 50 μM and 20 μM, respectively) (Table 1, Additional file 3: Figure S1). Since *B. burgdorferi* is a major human pathogen that has been reported in this tick, this data suggested the potential roles of HlDFS1 and HlDFS2 in controlling this pathogen in ticks. In addition, HlDFS1 inhibited the growth of fungus *C. albicans* with an MIC_90_ of 50 μM, while HlDFS2 did not impact the fungus growth. Both defensins showed no significant influence on vesicular stomatitis virus (VSV) replication, as determined by adding HlDFS1 and HlDFS2 into the media of 293 T cells infected with VSV-GFP virus (Additional file 4: Figure S2).

**Table 1:** Antimicrobial profile of HlDFS1 and HlDFS2

Strain	HlDFS1		HlDFS2	
	MIC_50_ (μM)	MIC_90_ (μM)	MIC_50_ (μM)	MIC_90_ (μM)
Gram-positive bacteria				
*Bacillus pumilus* (CMCC63202)	No effect	No effect	No effect	No effect
*Staphylococcus aureus* (CMCC26003)	10	50	50	> 50
*Micrococcus luteus* (CMCC28001)	5	10	1	1
*Mycobacterium bovis*	0.2	0.5	0.5	0.5
Gram-negative bacteria				
*Salmonella typhimurium* (CVCC542)	No effect	No effect	No effect	No effect
*Pseudomonas aeruginosa* (CVCC2000)	No effect	No effect	No effect	No effect
*Escherichia coli* (CMCC44103)	5	5	1	1
*Borrelia burgdorferi* (297-GFP)	50	50	5	20
Fungi				
*Candida albicans* (CAU0037)	50	> 50	No effect	No effect

*Abbreviations*: MIC_50_ and MIC_90_: minimum inhibitory concentration required to inhibit the growth of 50% (MIC_50_) or 90% (MIC_90_) of organisms. Determination of MICs was performed at least three times in triplicates

### HlDFS1 and HlDFS2 inhibit the growth of antibiotic resistant bacteria

As drug resistance is a growing public health concern, we explored the role of HlDFS1 and HlDFS2 on eleven clinical isolated antibiotic resistant bacteria. As shown in Table 2, both HlDFS1 and HlDFS2 had significant inhibitory activities against various antibiotic resistant Gram-positive and Gram-negative bacteria. For example, HlDSF1 and HlDFS2 inhibited antibiotic resistant *Staphylococcus epidermidis* strain No. 527 at an MIC_50_ of 2 μM and MIC_90_ of 2 μM, respectively. HlDSF1 and HlDFS2 also killed drug-resistant Gram-negative *Acinetobacter baumannii* strain No. 531 at an MIC_50_ of 5 μM and 10 μM, respectively. These data indicated that HlDFS1 and HlDFS2 have the ability to inhibit some antibiotic resistant bacteria and can be potential candidates for clinical application.

**Table 2:** Antimicrobial activity of HlDFS1 and HlDFS2 against antibiotic resistant bacteria

Strain	HlDFS1		HlDFS2	
	MIC_50_ (μM)	MIC_90_ (μM)	MIC_50_ (μM)	MIC_90_ (μM)
Gram-positive bacteria				
*Staphylococcus aureus* (No. 570)	50	> 50	> 50	> 50
*Staphylococcus epidermidis* (No. 526)	20	50	20	50
*Staphylococcus epidermidis* (No. 527)	5	50	2	2
*Staphylococcus epidermidis* (No. 532)	50	> 50	No effect	No effect
Gram-negative bacteria				
*Acinetobacter baumannii* (No. 531)	5	50	10	20
*Acinetobacter baumannii* (No. 546)	50	> 50	50	> 50
*Enterobacter aerogenes* (No. 516)	50	> 50	> 50	> 50
*Escherichia coli* (No. 572)	> 50	> 50	50	> 50
*Escherichia coli* (No. 582)	50	> 50	> 50	> 50
*Klebsiella pneumoniae* (No. 570)	No effect	No effect	No effect	No effect
*Klebsiella pneumoniae* (No. 593)	> 50	> 50	No effect	No effect

*Abbreviations*: MIC_50_ and MIC_90_: minimum inhibitory concentration required to inhibit the growth of 50% (MIC_50_) or 90% (MIC_90_) of organisms. Determination of MICs was performed at least three times in triplicates

### HlDFS1 and HlDFS2 are not hemolytic and cytotoxic to mammalian cells

**Fig. 3:**
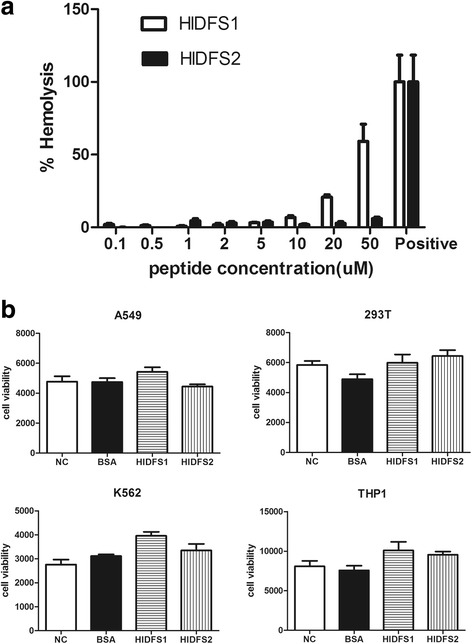
Hemolytic and cytotoxic assay of HlDFS1 and HlDFS2. **a** HlDFSs showed no significant hemolytic effect at concentrations of 10 μM (HlDFS1) and 50 μM (HlDFS2). Red blood cells were lysed by 0.4% TritonX 100 and loaded as positive controls. **b** HlDFS1 and HlDFS2 showed no detectable cytotoxic effect on mammalian cell line A549, 293 T, K562 and THP1 at a concentration of 20 μM. Cell survival rates were measured by using Cell Viability Assay (Promega) according to the manufacturer’s instruction. Results are expressed as the mean ± SEM. The representative results from at least three independent experiments are shown

To test whether HlDFS1 and HlDFS2 have cytotoxic effect on mammalian cells, we measured the hemolysis efficiency of both peptides in concentrations effective at killing Gram-positive bacteria. The results showed that both HlDFS1 and HlDFS2 are harmless to human erythrocytes in concentrations of up to 10 μM (Fig. 3a). Furthermore, HlDFS1 and HlDFS2 showed no detectable cytotoxicity to multiple mammalian cell lines, including A549, 293 T, K562 and THP1 cells (Fig. 3b).

### HlDFS1 and HlDFS2 significantly protect mice against lethal bacterial infection

**Fig. 4:**
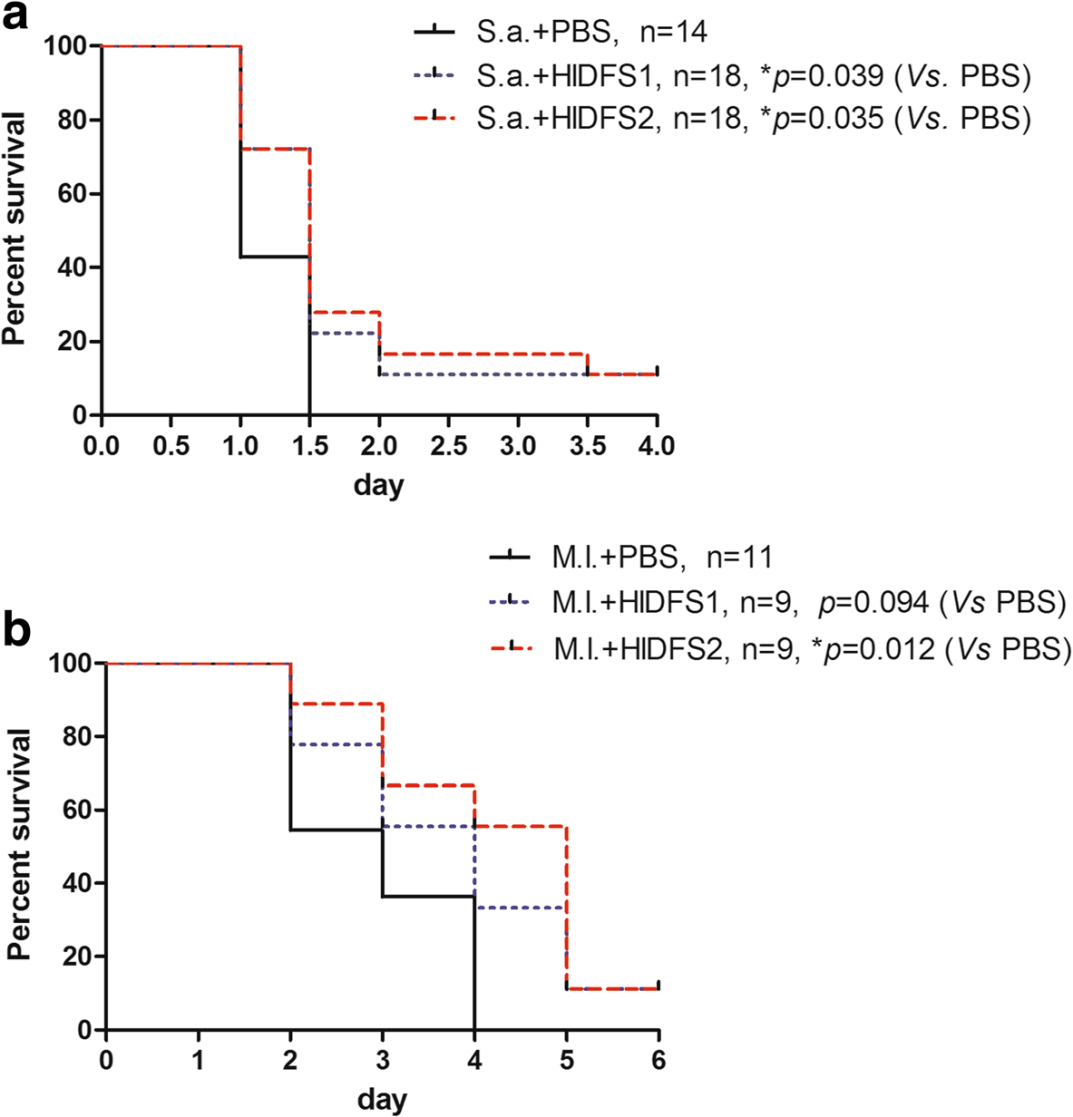
HlDFS1 and HlDFS2 significantly protect mice against lethal infection of *S. aureus* and *M. luteus*. Mice were infected with lethal doses of *M. luteus* (**a**) (1 × 10^6^ *cfu* per C3H/HeJ mouse) and *S. aureus* (**b**) (1 × 10^8^ *cfu* per C57/BL6 mouse) by i.p. injection. Six hours after infection, mice were treated by i.p. injection of HlDFS1 (100 μg/mouse), HlDFS2 (100 μg/mouse) or saline as controls. Mice were monitored daily for survival and symptoms of disease. Log-rank (Mantel-Cox) tests were used for these survival data

Since HlDFS1 or HlDFS2 shows significant bactericidal activity against Gram-positive bacteria *M. luteus* and *S. aureus*, we tested the antimicrobial activity of HlDFS1 or HlDFS2 in vivo using mouse infection models. The results suggested that both HlDFS1 and HlDFS2 could significantly extended survival time of mice infected by *S. aureus* from 1.5 days to more than 4 days (Fig. 4a). Meanwhile, HlDFS1 and HlDFS2 could extend survival time of mice infected by *M. luteus* from 4 days to more than 6 days (Fig. 4b). These results indicated that HlDFS1 and HlDFS2 had therapeutic efficacy on mice infected by bacteria.

## Conclusions

In conclusion, two functional defensin genes, HlDFS1 and HlDFS2, were identified from *H. longicornis* of China. Both two defensin isoforms possess bactericidal properties against selective Gram-positive and Gram-negative bacteria as well as antibiotic resistant *S. epidermidis* and *A. baumannii*. Most importantly, HlDFS1 and HlDFS2 had therapeutic efficacy on mice challenged by lethal bacterial infection. These data suggest that HlDFS1 and HlDFS2 could be safely used in mammalian systems as a potential antimicrobial reagent against various bacteria and other pathogens.

## Additional files

Additional file1: Table S1. Primer sequences for the RT-PCR assay. (DOCX 14 kb)

Additional file 2: Table S2. The antibiotic resistant information of selective drug resistant bacteria strains. (XLSX 15 kb)

Additional file 3: Figure S1. HlDFS1 and HlDFS2 inhibited the growth of *B. burgdorferi.* Quantitative RT-PCR (**a**) and electrophoresis results (**b**) for *B. burgdorferi flaB* gene in DNA samples of spirochete culture. (**c**) Fluorescence microscopy analysis of GFP signals from *B. borgdorferi* GFP-297 strains. *B. borgdorferi* GFP-297 is an engineered strain steadily expression GFP protein on the surface of spirochetes. (TIFF 2113 kb)

Additional file 4: Figure S2. HlDFS1 and HlDFS2 showed no significant antiviral activity against VSV. 293 T cells were infected with VSV-GFP virus at an MOI = 1 (VSV-GFP virus that expresses GFP as a non-structural protein was provided by Dr. Chunsheng Dong, Soochow University). 20 μM HlDFS1 and HlDFS2 or BSA controls were added into the cell culture. 12 h post-infection, VSV-GFP replication were visualized by GFP signal under the microscope. (TIFF 7201 kb)

## Abbreviations

AMP: Antimicrobial peptide
CVCC: China Veterinary Culture Collection Center
GFP: green fluorescent protein
HlDFS1: *Haemaphysalis longicornis* defensin 1
HlDFS2: *Haemaphysalis longicornis* defensin 2
i.p.: intraperitoneal
ORF: open reading frame
RT-PCR: reverse transcription polymerase chain reaction
