# Supplementary material for: Functional characterization of two defensins, HlDFS1 and HlDFS2, from the hard tick Haemaphysalis longicornis
Source: Parasit Vectors. 2017 Oct 2;10:455. doi: 10.1186/s13071-017-2397-9 (PMC5625651; doi:10.1186/s13071-017-2397-9)
Supplement: Supplementary file 1 — Primer sequences for the RT-PCR assay. (DOCX 14 kb) [file 13071_2017_2397_MOESM1_ESM.docx]

| **Primer name** | **Primer sequence** |
| --- | --- |
| HlDFS1 Forward | AAAGGAGCAGTCACTGAAGAG |
| HlDFS1 Reverse | ACGGACGCAAGTGCAGGTGAG |
| HlDFS2 Forward | GAGGAGGAAAGCGAAGTGGCA |
| HlDFS2 Reverse | GTTTCTATAGCAGGTGCAGGT |
| *B. burgdoreferi flaB* Forward | AAGAGCTTGGAATGCAGCCT |
| *B. burgdoreferi flaB* Reverse | AGCAGTTTGAGCTCCCTCAC |
| Tick Actin  Forward | GCCCTGGACTTCGAGCAGGA |
| Tick Actin  Reverse | CACGTCGCACTTCATGATGG |

**Additional file 1: Table S1** Primer sequences for the RT-PCR assay
