# Supplementary figures and images for: Functional characterization of two defensins, HlDFS1 and HlDFS2, from the hard tick Haemaphysalis longicornis
Source: Parasit Vectors. 2017 Oct 2;10:455. doi: 10.1186/s13071-017-2397-9 (PMC5625651; doi:10.1186/s13071-017-2397-9)

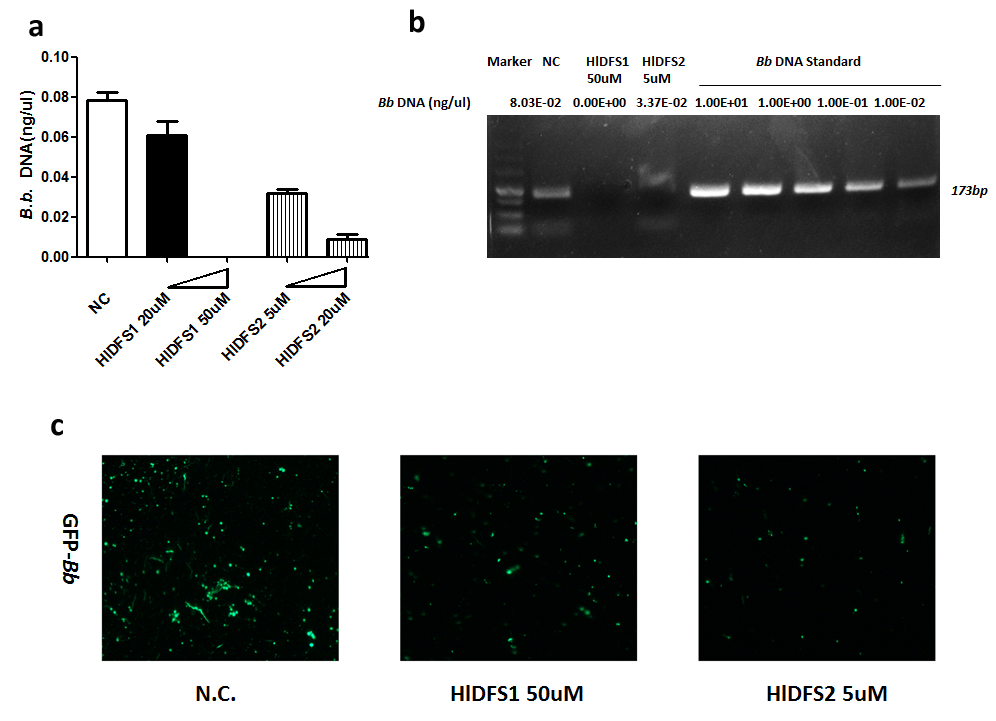

Supplement: Supplementary file 3 — HlDFS1 and HlDFS2 inhibited the growth of B. burgdorferi. Quantitative RT-PCR (a) and electrophoresis results (b) for B. burgdorferi flaB gene in DNA samples of spirochete culture. (c) Fluorescence microscopy analysis of GFP signals from B. borgdorferi GFP-297 strains. B. borgdorferi GFP-297 is an engineered strain steadily expression GFP protein on the surface of spirochetes. (TIFF 2113 kb) [file 13071_2017_2397_MOESM3_ESM.tif]

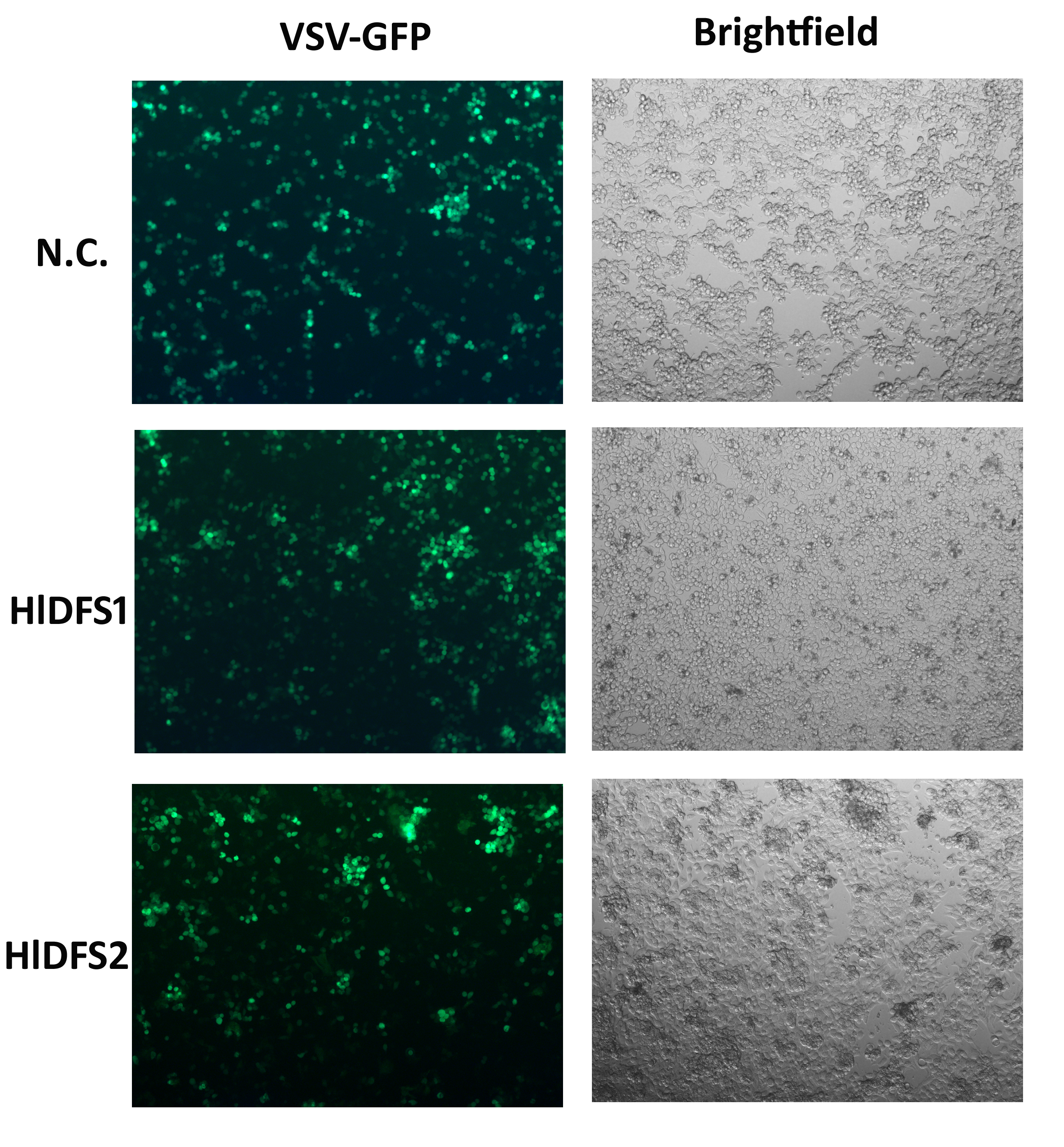

Supplement: Supplementary file 4 — HlDFS1 and HlDFS2 showed no significant antiviral activity against VSV. 293 T cells were infected with VSV-GFP virus at an MOI = 1 (VSV-GFP virus that expresses GFP as a non-structural protein was provided by Dr. Chunsheng Dong, Soochow University). 20 μM HlDFS1 and HlDFS2 or BSA controls were added into the cell culture. 12 h post-infection, VSV-GFP replication were visualized by GFP signal under the microscope. (TIFF 7201 kb) [file 13071_2017_2397_MOESM4_ESM.tif]
